# Antibiotic resistance profiles and activity of clove essential oil (*Syzygium aromaticum*) against *Pseudomonas aeruginosa* isolated of canine otitis

**DOI:** 10.14202/vetworld.2022.2499-2505

**Published:** 2022-10-30

**Authors:** Larissa Vieira Costa, Janaina Marcela Assunção Rosa Moreira, Isabela de Godoy Menezes, Valéria Dutra, Arleana do Bom Parto Ferreira de Almeida

**Affiliations:** 1 Program of Postgraduate in Veterinary Sciences, Faculty of Veterinary Medicine, Federal University of Mato Grosso, Cuiabá – Mato Grosso, Brazil; 2 Medical Sciences College, Federal University of São Paulo, São Paulo, Brazil; 3 Veterinary Hospital, Faculty of Veterinary Medicine, Federal University of Mato Grosso, Cuiabá – Mato Grosso, Brazil

**Keywords:** essential oil, multidrug resistance, *Pseudomonas aeruginosa*, susceptibility

## Abstract

**Background and Aim:**

*Pseudomonas aeruginosa* is often isolated from acute and chronic otitis and deep pyoderma in dogs. The increase in bacterial resistance to antibiotics induced the need for alternative therapies to treat infections, with an emphasis on essential oils (EOs). This study aimed to investigate clove oil’s *in vitro* bactericidal action as a therapeutic alternative against strains of *P. aeruginosa* isolated from canine otitis.

**Materials and Methods:**

The antibacterial activity of clove oil was evaluated by determining the minimum inhibitory concentration (MIC) and minimum bactericidal concentration (MBC) using the broth microdilution technique in 96-well plates. Serial concentrations of 10–0.31% of the oil were used, equivalent to 104.5–3.26 mg/mL. The susceptibility of isolates to different classes of antibiotics was determined by the disk diffusion technique using 20 antibiotics belonging to eight classes. Isolates resistant to at least one antibiotic of three different classes were considered multidrug-resistant (MDR).

**Results:**

A high occurrence of resistance was observed for three antibiotics belonging to the cephalosporin classes (cefadroxil, cephalexin, and ceftriaxone), namely, sulfamethoxazole + trimethoprime, doxycycline, and enrofloxacin. The lowest resistance rates were observed for meropenem (4.88%), amikacin (12.20%), and tobramycin (12.2%). All isolates were susceptible to clove oil with an equivalent MIC and MBC from 3.26 to 6.53 mg/mL. Eugenol was the major component of the oil.

**Conclusion:**

Clove EO was effective against MDR strains of *P. aeruginosa*, indicating an alternative for developing an efficient and low-cost antimicrobial agent to treat canine otitis.

## Introduction

The increase in infections caused by multidrug-resistant (MDR) bacteria has culminated in a global public health crisis, increasing morbidity and mortality rates and making the diagnosis and treatment of these infections challenging [1]. Bacterial resistance is an emerging problem in veterinary medicine, mainly due to the excessive use of antibiotics [2]. Among MDR bacteria, *Pseudomonas aeruginosa* is a ubiquitous gram-negative rod bacteria considered one of the most important opportunistic pathogens that cause nosocomial infections, especially in immunocompromised patients [3]. *Pseudomonas aeruginosa* presents multi-resistance to several classes of antibiotics and can acquire resistance during treatment [4]. This bacterium is often isolated from acute and chronic otitis and deep pyoderma in dogs, making it a potential reservoir of zoonotic MDR bacteria when handled by their owners [5].

There is an urgent requirement to produce new antibiotics because low efficacy has already been reported for the new generations of these agents. In 2017, the World Health Organization published a list of priorities for the production of new antibiotics, and *P. aeruginosa* resistant to carbapenem antibiotics was at the top of the urgency list, classified as “critical” [2, 6]. Thus, there is a growing need for alternative therapies to treat infections, with an emphasis on essential oils (EOs) [7, 8]. Clove EO (*Syzygium aromaticum*) has been studied for its therapeutic potential as an anti-inflammatory, antioxidant, antiviral, antifungal, antimicrobial, analgesic, anticarcinogenic, antidiabetic, and anesthetic agent [9–11]. Studies have demonstrated its effectiveness against bacteria of great relevance to public health, such as *P. aeruginosa*, *Acinetobacter* spp., *Proteus vulgaris*, *Enterobacter cloacae*, *Escherichia coli*, *Salmonella choleraesuis*, *Salmonella enterica*, *Bacillus cereus*, *Serratia marcescens*, *Shigella flexner*, *Listeria monocytogenes*, *Typhimurium* spp., and *Staphylococcus aureus* [12–14].

The previous study evaluated the effectiveness of EOs on bacterial isolates from dogs, and there is little research on pathogens obtained from canine otitis [15]. This study aimed to investigate the antibacterial effect of clove EO against *P. aeruginosa* strains isolated from canine otitis and the resistance profile of the isolate to several classes of antibiotics.

## Materials and Methods

### Ethical approval

This research was approved by the Ethics Committee on Animal Use of the Federal University of Mato Grosso under number 23108.221438/2017-83.

### Study period and location

The study was conducted from May 2020 to May 2021 at the Small Animal Medical Clinic and Laboratory of Microbiology and Molecular Biology of the Veterinary Hospital of Federal University of Mato Grosso, Cuiabá, Mato Grosso, Brazil.

### Clove EO and chromatographic evaluation

Clove EO (*S. aromaticum*) processed by the steam distillation technique was purchased commercially from company QUINARÍ^®^, Ponta Grossa, Paraná, Brazil. Chemical composition analysis was carried out in the chromatography laboratory of the Department of Chemistry of the Federal University of Minas Gerais. Quantitative evaluation was performed using high-resolution gas chromatography (GC)-flame-ionization detection and qualitative analysis by GC coupled to a mass spectrometer.

### Bacterial isolament and identification *P. aeruginosa*

In this study, 50 isolates of *Pseudomonas* spp. collected from dogs with an established diagnosis of bacterial otitis externa were obtained and processed at the Laboratory of Microbiology and Molecular Biology of the Veterinary Hospital of Federal University of Mato Grosso. Briefly, the otological swabs were seeded on 8% sheep blood agar (Sigma-Aldrich, Darmstadt, Germany) and MacConkey agar (Neogen Corporation, São Paulo, Brazil) under aerobic conditions, incubated at 37°C for up to 72 h and characterized morphologically and biochemically as described by Quinn *et al*. [16]. The isolates were pelleted, transferred to glycerol, and stored at −86°C until further processing.

To identify the *P. aeruginosa* species, genomic DNA was extracted from all isolates using the phenol-chloroform extraction protocol [17]. The extracted DNA was subjected to a polymerase chain reaction (PCR) targeting the 16 S Ribosomal Ribonucleic Acid (16S rRNA) gene. The oligonucleotide sequences used were: 27 Forward: AGA GTT TGA TCC TGG CTC AG [18] and 1492 Reverse: GGT TAC CTT GTT ACG ACT T [19] amplifying a fragment of 1512 base pairs.

The amplification products obtained were purified using the Illustra GFX PCR DNA and Gel Band purification kit (GE Healthcare Life Sciences, Buckinghamshire, UK) and sequenced using the ABI 3500 Genetic Analyzer automatic sequencer (Applied Biosystems, Foster City, CA, USA). The sequences were compared with those in the GenBank database using BLAST on the NCBI server (http: www.ncbi.nlm.nih.gov/BLAST). The isolates that obtained at least 97% identity with *P. aeruginosa* were selected to perform the susceptibility test [20].

### Antibiotic resistance profile

The isolated strains were subjected to antimicrobial susceptibility testing using the agar gel disk diffusion method [21], following the standards established by the Clinical Laboratory Standards Institute and according to the manual M100-S25 [22]. The antimicrobials tested were: Aminoglycosides: Gentamicin (10 μg), tobramycin (10 μg), amikacin (30 μg), neomycin (30 μg); carbapenems: Meropenem (10 μg), imipenem (10 μg); cephalosporins: ceftazidime (30 μg), cefepime (30 μg), cephalexin (30 μg), cefadroxil (30 μg), ceftriaxone (30 μg); fluoroquinolones: enrofloxacin (5 μg), ciprofloxacin (5 μg), levofloxacin (5 μg), marbofloxacin (5 μg); penicillins: amoxicillin with clavulanic acid (30 µg), piperacillin+tazobactan (30/6 µg); monobactams: aztreonan (30 μg); sulfonamides: sulfamethoxazole+trimetropine (25 μg); and tetracyclines: doxycycline (30 μg) [23–25]. The isolates were classified as sensitive or resistant, and the intermediates were considered resistant during analysis. The strain was considered MDR when it showed resistance to an antibiotic of three or more classes [26]. Furthermore, the multiple resistance index proposed by Krumperman [27] was used.

### Antimicrobial activity of clove oil (*S. aromaticum*)

The antimicrobial activity of clove oil was determined by obtaining the minimum inhibitory concentration (MIC) and minimum bactericidal concentration (MBC) using the broth microdilution technique in 96-well polystyrene microplates, as described by Santos *et al*. [28]. Initially, the oil was diluted in sterile distilled water in a proportion of 4.5 mL of water + 0.5 mL of EO + 0.05 mL of Tween 80, obtaining a 10% stock solution. To evaluate the MIC, the following serial dilutions of the oils were made: 10%, 5%, 2.5%, 1.25%, 0.62% to 0.31%, equivalent to concentrations of 104.5 mg/mL, 52.25 mg/mL, 26.12 mg/mL, 13.06 mg/mL, 6.53 mg/mL, and 3.26 mg/mL. Afterward, 10 μL of the bacterial suspension adjusted to 0.5 on the McFarland scale (1.5 × 10^8^ colony-forming unit [CFU]/mL) was inoculated into the wells of the microplates already prepared with Mueller-Hinton (MH) medium. Finally, 100 μL of the serially diluted oil was added.

The plates were covered and incubated at 37°C for 24 h, with subsequent visual readings to determine the MIC, which corresponded to the lowest concentration that completely inhibited bacterial growth. During the assay, a positive (MH broth + standard strain + gentamicin), negative or sterile (MH medium only), and growth controls (MH medium + standard strain) were used. Serial dilutions of gentamicin (32–1 µg/mL) were used, equivalent to 10–0.31% of the antibiotic. A standard strain of *P. aeruginosa* (ATCC 27853) was used for quality control of the test. All experiments were performed in triplicate.

To determine the minimum bactericidal concentration (MBC), a 10 µL aliquot of the dilutions corresponding to the MIC and two dilutions greater than this was added to seed MH medium and incubated at 37°C for 24 h to observe colony growth. Minimum Bactericidal Concentration was considered the lowest concentration that prevented the visible growth of bacteria or allowed the growth of up to three CFU. These concentrations were considered bactericidal, and concentrations with more than three CFUs were considered bacteriostatic [29].

### Statistical analysis

The data were described and tabulated in Excel spreadsheets to obtain the mean and standard deviation measurements. The means of minimum inhibitory and bactericidal concentrations of clove EO and gentamicin were compared using Student’s t-test, with a significance level of 5%, using the Prism 5 software (GraphPad).

## Results

Among the 50 isolates of *Pseudomonas* spp. identified in the biochemical tests, 41 were confirmed as *P. aeruginosa* by sequencing. The other isolates were identified *as Pseudomonas stutizeri* (2), *Achromobacter xylosoxidans* (1), and *Klebisiella oxytoca* (1). The other isolates showed low similarity with *P. aeruginosa*. Susceptibility tests revealed high resistance rates among the evaluated isolates (Table-1). In total, 20 antibiotics belonging to eight classes were tested. Regarding the susceptibility profile, the highest resistance rates were observed for cephalosporins (cefadroxil 97.56%, cephalexin 97.56%, and ceftriaxone 92.69%), sulfamethoxazole+trimetropine (97.56%), doxycycline (95.12%), and enrofloxacin (78.05%). The lowest resistance rates were observed for meropenem (4.88%), amikacin (12.20%), and tobramycin (12.2%). None of the isolates was resistant or sensitive to all antimicrobials.

**Table-1:** Susceptibility to antibiotics of *Pseudomonas aeruginosa* isolates from canine otitis.

Antimicrobial class	Resistant n (%)	Sensitive n (%)	Total (%)
Aminoglycosides			
Amikacin	05 (12.2)	36 (87.80)	41 (100)
Gentamicin	12 (29.27)	29 (70.73)	41 (100)
Neomycin	20 (48.78)	21 (51.22)	41 (100)
Tobramycin	05 (12.2)	36 (87.80)	41 (100)
Beta-lactamics (Carbapenemics)			
Imipinem	11 (26.83)	30 (73.17)	41 (100)
Meropenem	02 (4.88)	39 (95.12)	41 (100)
Beta-lactamics (Cephalosporins)			
Cefadroxil	40 (97.56)	01 (2.44)	41 (100)
Cephalexin	40 (97.56)	01 (2.44)	41 (100)
Cefepime	15 (36.59)	26 (63.41)	41 (100)
Ceftazidime	08 (19.51)	33 (80.49)	41 (100)
Ceftriaxone	38 (92.69)	03 (7.32)	41 (100)
Beta-lactamics (Monobactamics)			
Aztreonam	27 (65.85)	14 (34.15)	41 (100)
Beta-lactamics (Penicillins)			
Piperacillin + tazobactam	12 (29.27)	29 (70.73)	41 (100)
Quinolones			
Ciprofloxacin	12 (29.27)	29 (70.73)	41 (100)
Enrofloxacin	32 (78.05)	09 (21.95)	41 (100)
Levofloxacin	17 (41.47)	24 (58.54)	41 (100)
Marbofloxacin	15 (36.59)	26 (63.41)	41 (100)
Sulfonamides			
Sulfamethoxazole + trimetropine	40 (97.56)	01 (2.44)	41 (100)
Tetracycline Doxycycline	39 (95.12)	02 (4.88)	41 (100)

Among the 41 *P. aeruginosa* isolates analyzed, 40 (97.56%) showed resistance to more than three classes of antibiotics and were classified as MDR bacteria. The values of the multi-resistance index varied from 0.05 to 0.9, with an average of 0.52.

The chromatographic analysis of the EO of clove (*S. aromaticum*) identified eugenol (85.2%) as the major compound, followed by β-caryophyllene (8.7%), α-humulene (2.2%), α-cubebene (1.4%), benzyl benzoate (0.7%), caryophyllene oxide (0.3), δ-cadinene (0.2%), and others (1.2%).

**Figure-1:**
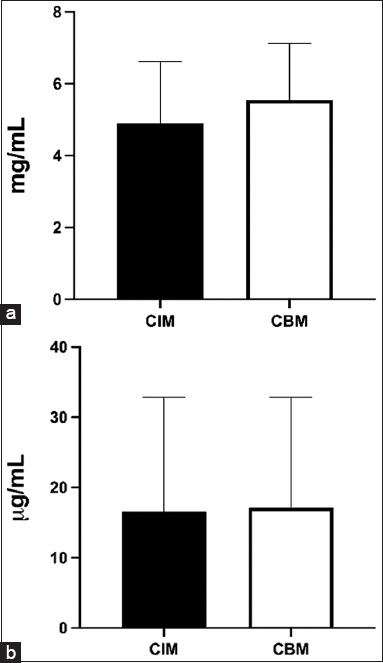
Comparison between the means and deviations of minimum inhibitory concentration and minimum bactericidal concentration of (a) clove essential oil and (b) gentamicin.

Clove EO showed antimicrobial activity against all ten selected MDR isolates of *P. aeruginosa*. Using the broth microdilution technique, the minimum inhibitory and MBCs were obtained, which varied from 3.26 mg/mL (0.31%) to 6.53 mg/mL (0.63%). Half of the isolates were resistant to gentamicin. There was no statistically significant difference between the mean MIC and MBC of clove EO (p > 0.05), as shown in Figure-1. Table-2 shows the mean and deviation values of the MIC and MBC of clove EO and gentamicin.

**Table-2:** Means and standard deviations of MIC and MBC of clove essential oil and gentamicin.

Analysis	Clove oil (mg/mL)		Gentamicin (µg/mL)	
	
	MIC	MBC	MIC	MBC
Mean	4.9	5.55	16.60	17.10
Standard deviations	1.72	1.58	16.24	15.74

MIC=Minimum inhibitory concentration, MBC=Minimum bactericidal concentration

## Discussion

The emergence of MDR strains is a major challenge in public health. *Pseudomonas aeruginosa*, a common bacterium in antibiotic-resistant hospital infections, is associated with treatment-refractory canine otitis media [30, 31]. In this study, the occurrence of MDR isolates was 97.56%, and more than 30% were resistant to carbapenem antibiotics, which are considered antipseudomonal antibiotics, an alarming finding pointed out by other authors [32, 33].

The highest resistance rates were in the beta-lactams class, particularly cephalosporins and penicillins. Cephalexin, cefadroxil, and amoxicillin associated with clavulanic acid, widely used in treating veterinary dermatoses, proved to be ineffective, corroborating the results of other studies [34, 35]. Carbapenem-resistant *P. aeruginosa* poses a global threat to public health, as this class of antibiotics represents the last-resort treatment in medicine. In this study, 31.71% of the isolates were resistant to the carbapenems tested. The low susceptibility of the isolates to anti-pseudomonal antibiotics, such as aztreonam, imipenem, ceftazidime, cefepime, and piperacillin associated with tazobactam, is alarming. It reflects the skillful defense developed by *P. aeruginosa* against these agents and the scarce remaining therapeutic resources [36, 37]. Another valuable finding was the greater susceptibility of the isolates to ceftazidime (Table-1) than to cefepime, as the latter is a fourth-generation cephalosporin.

Similarly, quinolones showed high resistance levels, including enrofloxacin (78.05%) and marbofloxacin (36.59%), both for veterinary use. Resistance is due to the excessive and often empirical use of these antibiotics and intrinsic resistance mechanisms [38–40]. Aminoglycosides are widely used as topical therapies in cases of canine otitis; however, they are often prescribed in the absence of a susceptibility test. The indiscriminate use of this class of antibiotics may have induced the high resistance observed in this and other studies [41]. Among the commercial optical formulations available in Brazil, gentamicin and neomycin showed significant resistance rates, corroborating other results [42]. Other studies have evaluated the high efficacy of aminoglycosides, possibly due to their lower use in other countries due to their toxicity [25, 43].

Considering the bacteria’s high resistance, especially *P. aeruginosa*, rational use of antibiotics and the development of new compounds are necessary. In this sense, clove EO stands out for its bactericidal potential, as proven in this and other studies [44, 45]. Other authors also pointed to eugenol as the major component in clove oil and attributed it as the main component responsible for cloves’ therapeutic and antimicrobial effects [11, 13, 46]. Beta-caryophyllene also demonstrated an *in vitro* inhibitory action against *S. aureus* strains [47]. In the chromatographic evaluation of this study, beta-caryophyllene was the second-highest component, and together with eugenol, they are responsible for the antibacterial action of clove oil [12].

Recent research claims that the antibacterial action of clove oil is due to the rupture of the bacterial cell membrane. The lipophilic properties induce the extravasation of cellular components leading to cell death [47]. Other authors have also pointed out that eugenol inhibits bacterial DNA synthesis, prevents replication, and inhibits bacterial quorum sensing and expression migration of virulence factors. In addition, it prevents biofilm formation, an important defense mechanism for the bacterium [45]. These mechanisms explain the bactericidal action of clove EO observed in this study. All isolates were susceptible to clove oil using the broth microdilution method in this study. There were no statistical differences between the minimum inhibitory and MBCs. Other studies have reported similar inhibitory concentrations against *P. aeruginosa* strains [48, 49], and greater susceptibility with lower MICs than those tested in this study [50].

Clove EO showed greater inhibitory action when compared to gentamicin. However, this comparison is not adequate, because Clove EO has several bioactive compounds in different concentrations. However, our objective was to demonstrate the results of Clove EO in relation to the antibiotic most used for the topical treatment of canine otitis. The mean MIC of clove oil was lower than that of gentamicin. This combined with the fact that 50% of the isolates were resistant to gentamicin, reinforces the data. The indiscriminate use of gentamicin and non-use of clove EO in treating otitis or skin diseases in dogs may have contributed to this result [42]. However, some studies describe total resistance to clove oil at various concentrations [28] and its effectiveness at only 100% concentration [51]. The differences in inhibitory concentrations in the different studies may be related to the methodology applied, the strains used, and intrinsic characteristics of the clove oil used, such as the variation in its components and ways of obtaining it, as well as intrinsic factors of the plant, such as climate, geographic location, and seasonality [47].

More research has been carried out on clove oil to search for natural compounds with antibacterial potential every year. However, few studies evaluate the efficacy of *P. aeruginosa* strains originating from infection in dogs; however, the greatest range of veterinary research focuses on *Staphylococcus* spp. and *Malassezia pachydermatis* [15]. Identifying the pathogen and its susceptibility profile aids in successfully treating the animal and reduces bacterial resistance to antibiotics [52, 53]. We also emphasize the relevance of research, such as the present one, which provides data that can be used in the future to produce new drugs necessary for the treatment of veterinary patients. The results of this study reinforce the conscious use of antimicrobials and point favorably to the antibiotic action of the tested oil.

## Conclusion

In this study, a high occurrence of antibiotic resistance was observed in *P. aeruginosa* isolates, reflecting the worldwide prevalence of bacterial multidrug resistance. Clove EO proved to be effective against MDR strains of *P. aeruginosa*, presenting MIC and MBC from 3.26 mg/mL-6.53 mg/mL, indicating the possibility of developing an efficient and low-cost antimicrobial agent as an alternative for treating canine otitis. However, *in vivo* studies are required to evaluate their effectiveness in practical applications, toxicity, and other pharmacological effects.

## Authors’ Contributions

LVC, ABPFA, and VD: Contributed to the conception and design of this study. LVC: Wrote the manuscript. IGM: Statistical analysis. LVC and JMARM: Performed the experiment. LVC and ABPFA: Reviewed the manuscript. All authors have read and approved the final manuscript.
